# The Effect of Perfectionism on Consumers’ Intentions to Purchase Imperfect Products

**DOI:** 10.3390/bs13030269

**Published:** 2023-03-18

**Authors:** Libin Chen, Guanhong Chen, Shuo Wang, Lin Jiang

**Affiliations:** 1 Business School, Beijing Technology and Business University, Beijing 100048, China; 2 School of Business, Renmin University of China, Beijing 100872, China

**Keywords:** perfectionism, imperfect products, dichotomous thinking, intolerance of uncertainty, purchase intention

## Abstract

Perfectionism is an important personality trait that affects people’s behavior, especially consumption behavior. In our study, we aimed to investigate whether perfectionists show different preferences in their consumption choices compared with non-perfectionists and to explore the potential psychological mechanisms mediating this effect. Through four studies, we found that perfectionists are less likely to buy imperfect products, including those that are close to expiry and that have defective functioning, flawed appearance, and incomplete after-sales service than non-perfectionists, and are more likely to avoid choosing imperfect products. In addition, we found a mediating effect of dichotomous thinking and intolerance of uncertainty on this effect to explain the behavioral preferences of perfectionists in their purchasing choices. Manufacturers and marketers can benefit from the results of this study by implementing targeted production requirements and marketing strategies based on the consumer behavior preferences of perfectionists.

## 1. Introduction

People naturally strive for the best and dream of attaining perfection [1]. Perfection is a widely respected virtue in today’s social value system [2] (pp. F1–F6), and the concept of perfectionism has penetrated our lives. Achieving perfection means a person receives more respect and appreciation, as well as more material rewards [3]. However, perfectionism can also be negatively viewed; relevant clinical evidence suggests that perfectionists are not actually perfect, and they commonly suffer from mental health problems such as anxiety and depression [4]. Therefore, the true nature of perfectionism, the causes of the emergence of our pursuit of perfection, and the impact it can have on our lives, including purchasing behavior, are areas that are worthy of our in-depth study.

Perfectionism is considered to be a personality trait in which one strives for high standards on task performance and is accompanied by a tendency toward critical self-assessment [5,6]. Because of perfectionists’ high standards and motivation to excel, they may look for or compare flaws and deficiencies in products with excessive care when shopping, so they can make accurate decisions and superior choices [1]. Perfectionists have higher requirements for product image integrity [7]. As such, we hypothesized that some differences may exist in the purchasing behavior between perfectionists and non-perfectionists for products that have some defects but do not affect their overall normal value, and whose quality and safety standards are within the acceptable range. We call this category of products imperfect products [8,9]. Hence, we aimed to discover whether perfectionists have different purchase intentions for imperfect products compared with non-perfectionists, and the psychological factors that influence the purchasing choice preferences of perfectionists.

Perfectionists tend to perform poorly when faced with difficult purchasing choice decision tasks as a result of their dichotomous thinking [1]. Seeing things as either “black or white” is a characteristic of dichotomous thinking [10], and combined with perfectionists’ high standards, whenever things are slightly flawed, dissatisfaction can be triggered in perfectionists, causing them to delay or abandon the original task. Perfectionists have a low tolerance for uncertainty [11], and imperfect products with broken packaging, immediate expiration dates, and defects all evoke the risk that consumers perceive the product as a threat to personal safety and health [12,13,14,15], so we speculated that the intolerance of uncertainty may also contribute to perfectionists’ preference to avoid purchasing imperfect products.

In this study, our main goal was to determine whether perfectionism significantly impacted consumers’ decisions to buy an imperfect product, and find out whether dichotomous thinking and intolerance of uncertainty are psychological mechanisms that can explain this effect. We aimed to examine the existence of a research framework showing that perfectionists (vs. non-perfectionists) are less likely to buy imperfect products (vs. perfect products) (nearing shelf life vs. not nearing shelf life; defective function vs. perfect function; flawed appearance vs. aesthetic appearance; incomplete after-sales service vs. complete after-sales service) and whether dichotomous thinking and intolerance of uncertainty play mediating roles. This study enriches the theoretical basis for perfectionism in consumer behavior, which can also provide relevant practical suggestions for marketers, retailers, and brands.

## 2. Theoretical Background

### 2.1. Imperfect Products

#### 2.1.1. Formal Imperfect Products

Imperfect or suboptimal products are products that deviate from the normal or optimal state, but the quality and safety standards of the product are within the acceptable range [8,9,16]. Imperfect products are judged based on having substantial variation from prototypicality for the following criteria: (a) appearance (e.g., in terms of weight, shape, or color) [13,17,18,19]; (b) date labeling (e.g., close to or beyond the best-before date) [20]; (c) packaging (e.g., torn bags and dented cans) [15]; and (d) function (e.g., low-quality versions of the product). From this, we can find that imperfection in products refers to formal imperfection.

First, regarding product appearance flaws, Grewal et al. [19] reported that consumers prefer to devalue unattractive products because of altered self-perceptions. In another study, imperfect produce increased purchase embarrassment and reduced purchase intention and retailer patronage intention [21]. Studies conducted from the perspective of product aesthetics showed that consumers perceive foods with classical aesthetic appearances to be healthier and show a higher willingness to pay for these than for ugly foods [22]. Aesthetically imperfect foods are more likely to evoke perceived risk, and thus, lower purchase intentions [12].

Studies on consumers’ willingness to buy imperfect products showed that consumers with high cognitive load perceive products with damaged outer surface packaging (such as broken express packages) as potentially dangerous and posing risks to personal health and safety. Accordingly, consumers with a high cognitive load have lower purchase intention for such imperfect products [15]. Another study on date labeling of products showed that 62% of consumers are more willing to buy products with the longest remaining shelf life [14], which indicated that a considerable number of consumers avoid buying products that are within their shelf life range but close to the end, as the safety and health risks of such goods are substantially increased for some vulnerable groups if the product is nearing the end of its shelf life [14].

Finally, in terms of product functionality, consumers perceive that the capability of a product is positively associated with the number of its features, and thus, infer that products with more functions provide more benefits [23]. In addition, studies show that perfectionist consumers evaluate products with perfect functions more highly than those with poor functions, because they believe that using products with perfect functions is a way to communicate social status to others [24].

Some suggestions show that consumers would be willing to buy imperfect products. The discrepancy in willingness to pay for unattractive versus attractive products can be reduced by altering the self-diagnostic signal of consumer choices and boosting consumers’ self-esteem [19]. Furthermore, labeling unattractive products as “ugly” combined with moderate price discounts [25], and transforming the physical state of imperfect products or anthropomorphizing unattractive products were proved to be successful strategies [26,27]. In conclusion, the above findings suggested that a segment of consumers with a low willingness to purchase imperfect products exists, but moderating factors alter the purchase decisions of this consumer group.

#### 2.1.2. Imperfect Product Service

Product hierarchy theory classifies products into five levels, where augmented product refers to the additional benefits and services that customers can obtain when they buy a product, which includes personalized and additional products that companies provide to customers [28] (pp. 141–145). After-sales service (ASS) is one of the forms of augmented products. After-sales service is mostly used to describe services that are delivered after the delivery of a certain commodity for the purpose of supporting consumer usage of the product throughout its life cycle [29]. After-sales service may include maintenance and replacement, spare parts supply, service assurance, customer relations, etc. [30]. Furthermore, companies focus more on keeping existing customers satisfied than on capturing new customers due to the relatively high customer acquisition costs [31]; thus, improving the quality of after-sale service can provide opportunities for enterprises to enhance their competitiveness and differentiation [32]. After-sales service is one of the important factors that affect customer satisfaction and customer loyalty [31].

Many researchers have examined the impact of after-sales service on consumer psychology and behavior. Pakdil et al. [33] identified the most important customer expectations about after-sales service quality as a reasonable warranty policy, immediate identification of product defects, and good customer service during the warranty period. In addition, Kumar et al. [34] regarded convenient operating hours, time taken in servicing, and personalized interaction with customers as the key elements determining the quality of after-sales service. The current studies defined after-sales service as another manifestation of the product and classified it into complete and incomplete according to the criterion of perfect or not. As the above speculations suggest, we examined whether a specific group of consumers whose attitude toward buying imperfect products is significantly different from other consumers, and determined the reasons affecting their attitude.

### 2.2. Perfectionism

Perfectionism was considered by Frost et al. [6] as a personality trait in which an individual strives for high standards of task performance and is accompanied by a tendency for critical self-assessment; it plays an important role in influencing human emotions and behaviors [35]. Kopylov [36] showed that having high standards for oneself and efforts to achieve high goals are the only criteria for self-recognizing perfectionists. As studies have progressed, a now large body of theoretical research and empirical evidence suggests that perfectionism is a multi-dimensional and complex structure, i.e., perfectionism has both positive and negative basic characteristics [37,38,39]. Although perfectionists strive for excellence, they also desperately try to avoid failure [40], and they will have excessive self-blame and self-doubt when not achieving their expected goals, which will lead to shame, anxiety, and other negative emotions [39,41]. The pursuit of excellence while avoiding failure shows the ambivalence of perfectionism and constitutes a basic personality trait of perfectionism.

In the field of marketing research, results on the direction of perfectionism are relatively scarce. Zhang [42] described the different motivators of perfectionists’ consumption behaviors through qualitative study methods, including (a) filling up the products they already owned, (b) completely breaking with imperfect products, and (c) having strict requirements on product hygiene. He [1] found that perfectionists, due to their dichotomous way of thinking, tend to stop investing and give up in advance when facing difficult consumption decisions because of the low possibility of achieving perfection, and their decision quality is lower than that of non-perfectionists. In combination with the objective situation, things are inevitably flawed or deficient, and perfectionists have harsh and high standards for things, so perfectionists generally do not consider things around them to be perfect and flawless. Imperfect objects disturb perfectionists’ anxiety, and their attention is, thus, drawn to imperfect objects [43]; consumers with higher perfectionism tend to have higher requirements for the integrity of product image [7], so they generally carefully look for product flaws in the shopping decision process to meet their perfection decision criteria.

As such, we proposed that when perfectionist consumers purchase products, they focus more on and care excessively about the imperfections in the form and service content of the product. Consequently, perfectionists are less willing to buy imperfect products than non-perfectionists. Therefore, based on the above analysis, the following hypotheses are proposed:

**Hypothesis 1 (H1).**  *Perfectionists are less likely to purchase formal imperfect products than non-perfectionists.*

**Hypothesis 2 (H2).**  *Perfectionists are less likely to purchase products with incomplete after-sales service than non-perfectionists.*

### 2.3. Dichotomous Thinking

Dichotomous thinking, also known as “black-or-white” thinking, is a type of cognitive distortion that is considered a strict information processing method [10]. These specific distortions in cognition affect how people think, feel, and behave [44]. Individuals with dichotomous thinking often see things from an extreme perspective, and they commonly evaluate experiences or things in terms of mutually exclusive, simple binary categories (e.g., good or bad; successful or failure; trustworthy or deceitful) rather than attributing experiences or things as falling along continua [45].

Dichotomous thinking is also present in the aspect of consumer behavior. Consumers often use categorical reasoning thinking to assess the value of a product, and they believe that a combination product containing both expensive and inexpensive items will have a lower value than the same expensive item sold separately [46]. In addition, consumers tend to clearly distinguish between positive and negative ratings when assessing customer review scores (e.g., five-star ratings), so they are unable to adequately consider extreme distributions, distort product evaluations, and make poor purchase decisions [47].

Perfectionism is strongly associated with dichotomous, rigid, and categorical methods of thinking [48,49,50]. Perfectionists also evaluate things or their own and others’ performance, usually in a simplistic dichotomous manner, and only make extreme black-or-white evaluations (thinking that something is performed successfully or not; thinking that products are perfect or imperfect). However, this black-or-white dichotomous mindset is considered a maladaptive trait, because it is determined based on a distorted and unrealistic perception of reality [46,51]. Although perfectionists are driven by high-performance standards, ironically, dichotomous thinking often prevents them from achieving these goals [1]. As He [1] found, one perfectionist consumer claimed, “Unless I have found the perfect gift, one that I am sure the recipient will like, then I would rather not give a gift at all”.

We suggest that perfectionistic consumers’ avoidance of imperfect products is influenced by dichotomous thinking. Because imperfection is a situation that perfectionists cannot accept, perfectionists, through their dichotomous thinking, categorize products with imperfection as products that do not meet their shopping standards, and thus, avoid buying imperfect products. Based on the above analysis, the following hypothesis is proposed:

**Hypothesis 3 (H3).**  *Dichotomous thinking plays a mediating role in the influence of perfectionism on the purchase intention of imperfect products.*

### 2.4. Intolerance of Uncertainty

Intolerance of uncertainty (IU) is a negative response of individuals in their cognition, emotions, and behaviors when faced with ambiguous stimuli or uncertain situations [52]—cognition, perceiving uncertainty as having negative consequences; emotions, showing frustration and stress in the face of uncertainty; behaviors, attempts to control the future and avoid present uncertainty [53], especially in response to ambiguous, novel, and unpredictable situations and events, are more prone to cognitive biases in perception, interpretation, and response [54,55,56].

High intolerance of uncertainty in an individual leads to assigning higher probabilities to poor expectations of future events, assigning higher implementation costs to innovative behaviors [57], and being more likely to negatively interpret information and develop anxiety once faced with information deficits [58]. Especially, consumer behavior literature points out that consumer uncertainty affects consumer behavior [59]. For example, Strong et al. [60] proposed that consumers’ uncertainty about their sense of self drives their interest in ancestral products (e.g., DNA services). In general, individual consumption behavior decisions do not blindly pursue the maximization of utility but are more inclined to avoid potential losses [61].

The perfectionist is typically an individual who is highly intolerant of uncertainty because uncertainty exacerbates the risk of imperfection, causing them to perceive increased distress, which in turn leads to a decrease in their tolerance of uncertainty [62,63]. The perfectionist is convinced that not achieving flawlessness will result in deleterious consequences and portend future failures [64], whereas achieving flawlessness provides momentary relief [65]. As such, the perfectionist seeks a state of perfection that does not allow for half-hearted flaws, incompleteness, or uncertainty. Markovi [11] found that negative perfectionistic individuals show more powerlessness and increased psychological distress toward uncertainty, and increased psychological distress, which makes them less tolerant of uncertain events, so they show a high intolerance of uncertainty.

In summary, broken and distorted external packaging, approaching the shelf life end date, and the deformed, defective, and unaesthetic value of imperfect products all evoke the risk that consumers perceive the product as a threat to their personal safety and health [13]. In addition, we speculate that for perfectionists who find the after-sales service of this product to be incomplete and inadequate or who find the product function to be defective, the uncertainty of their perceived future use of the product is also increased, aggravating the uncertainty of a satisfactory purchase outcome. For perfectionists, due to their personality traits of not being able to tolerate uncertainty, we hypothesize that they will develop avoidance behavior toward purchasing imperfect products to ensure that the uncertain event will not occur. Based on the above analysis, the following hypothesis is proposed:

**Hypothesis 4 (H4).**  *Intolerance of uncertainty plays a mediating role in the influence of perfectionism on the purchase intention of imperfect products.*

## 3. Study 1

In Study 1, we first focused on whether perfectionists were less willing to buy imperfect products and choose products nearing their shelf lives, one of the imperfect product dimensions, as the experimental material. We argue that the perceived consumption risk posed to consumers by products nearing the end of their shelf lives is intolerable to perfectionists and does not meet the strict selection criteria of perfectionists, so perfectionists develop avoidance behavior toward products nearing their end of shelf lives.

### 3.1. Method

#### 3.1.1. Participants

For Study 1, we recruited 140 undergraduates majoring in marketing at a university in Beijing as our participants, of which 55.71% were men and 44.29% were women, and their average age was 20.58 years. We informed the subjects that all the data we collected would be confidential and used only for academic research, but we did not inform them of the real purpose of the study to avoid bias in their answers. After completing the study, they were paid RMB 1.

#### 3.1.2. Procedure and Stimuli

Study 1 consisted of a 2 (perfectionist vs. non-perfectionist) × 2 (not nearing shelf life vs. nearing shelf life) between-subjects design. In Study 1, we first asked subjects to read a shopping situation: “Suppose you are choosing a food product in a retail supermarket and you see a carton of yogurt in the dairy section. The appearance of the product and its packaging are shown in the picture”. The picture of the yogurt used in the study is the same for both groups, and the yogurt box is labeled with the word “yogurt” and the words “shelf life 21 days” on the outer package. The picture of the yogurt box received by the perfect group had the words “20 days left to expiration” written underneath it; the picture of the yogurt box received by the imperfect group had the words “3 days left to expiration” written underneath it.

#### 3.1.3. WTP

Regarding the measurement of the willingness to pay, we used the method of Jensen and Drozdenko [66], in which the subjects were asked to answer the question: “Please give the lowest acceptable discount you would like to buy this product in the range of 0–100%” to measure their willingness to purchase the study product, which, thus, reflects their decision preferences (0% means they want to buy the product even if there is no discount; 100% means they do not want to buy the product even if it is completely free).

#### 3.1.4. Perfectionism

Regarding the measurement of perfectionist personality, we followed the perfectionist scale developed by Kopalle and Lehmann [67] based on the studies of Frost et al. [6] and Hewitt and Flett [68], using eight items to assess perfectionist personality, including “I get mad at myself when I make mistakes”, “It is very important for me to be right”, and “Little errors bother me a lot”. We used a seven-point Likert scale (“1” for strongly disagree and “7” for strongly agree). We classified the subjects as perfectionists and non-perfectionists according to their median scores.

### 3.2. Results

#### 3.2.1. Manipulation Check

We conducted a pretest study before Study 1, in which we measured whether subjects perceived the product in the study to be close to shelf life end, and the results of an independent samples T-test revealed that subjects significantly perceived yogurt with 3 days left to expire to be closer to shelf life end than yogurt with 20 days left to expire (M_20 days_ = 1.80, SD = 1.126 vs. M_3 days_ = 6.37, SD = 0.669, F (1, 58) = 364.569, *p* < 0.001).

#### 3.2.2. Reliability of Scale

Then, we examined the reliability of the scale of perfectionism (Cronbach’s alpha = 0.872, with 95% confidence intervals), which means that the analysis based on this scale is highly credible.

#### 3.2.3. WTP

**Figure 1:**
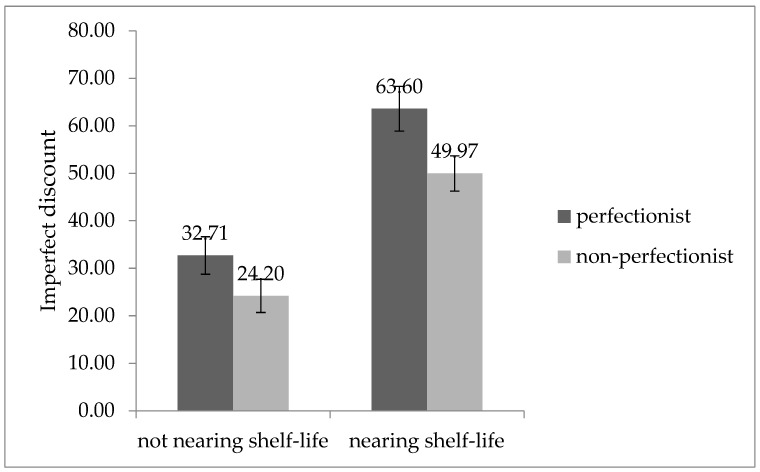
Willingness to pay for products nearing shelf life end or not according to perfectionists or non-perfectionists.

The analysis of variance (ANOVA) results in Figure 1 showed that perfectionism had a significant effect on consumers’ willingness-to-pay (F (1, 136) = 7.695, *p* = 0.006 < 0.01). Specifically, consumers with perfectionism were significantly less likely to purchase imperfect products nearing the end of shelf life than those without perfectionism (M_perfectionist_ = 63.60, SD = 27.819 vs. M_non-perfectionist_ = 49.97, SD = 21.974, F (1, 68) = 5.173, *p* = 0.026 < 0.05). In contrast, we found no significant difference between consumers with and without perfectionism in terms of willingness to pay for products not nearing shelf life end (M_perfectionist_ = 32.71, SD = 23.350 vs. M_non-perfectionist_ = 24.20, SD = 20.695, F (1, 68) = 2.606, *p* = 0.111 > 0.05).

### 3.3. Discussion

Different types of consumers have different attitudes toward the purchase of imperfect products, and we found that perfectionism significantly affected consumers’ attitudes toward the purchase of imperfect products. We tested this hypothesis in Study 1, where the main effect of H1 that we proposed was present. In Study 1, we measured the willingness to pay of perfectionists and non-perfectionists for a product either nearing shelf life end or not. By compiling and analyzing the obtained data, we found that consumers with perfectionist tendencies were more willing to avoid purchasing products nearing shelf life as formally imperfect products compared with those without perfectionist tendencies, but we observed no significant difference in their willingness to purchase products not nearing shelf life end.

## 4. Study 2

The purpose of Study 2 was to verify H1 and the mediating effect of H3: we choose another dimension of the formal imperfect product, function, as the experimental material for Study 2 to examine the robustness of H1 from another perspective, as well as to verify the influence of dichotomous thinking as a mediating factor. We argue that functional imperfections in products trigger the dichotomous thinking of perfectionists and that products lacking in functionality do not meet the higher purchase criteria that perfectionists presuppose in their minds, resulting in purchase avoidance behavior.

### 4.1. Method

#### 4.1.1. Participants

We completed this study with a class at a university in Beijing, with 144 participants in total, of which 6 questionnaires were considered invalid due to grouping conditions. Thus, the number of valid questionnaires was 138 (95.8%), of which 69.57% were completed by women and 30.43% by men. After completing the study, we paid the participants RMB 1.

#### 4.1.2. Procedure and Stimuli

Study 2 consisted of a 2 (perfectionist vs. non-perfectionist) × 2 (perfect function vs. defective function) between-subjects design. In Study 2, we first asked subjects to read a shopping situation: “Suppose you are planning to buy a desk lamp for your daily study and work use. The product features are described as follows”. We randomly divided the subjects into two groups: the perfect function group and the lack of function group, in which the perfect function group received the following description: “Infinitely dimmable (from dark to light, achieved by moving the touch slider; the darker to the left, the brighter to the right); with three color temperatures; multi-angle lighting; no blue light hazards, no visual flash; the product price of RMB 75”. The lack of function group received the following description: “non-adjustable brightness (only “on” and “off”); non-adjustable color temperature; multiangle lighting; no blue light hazards, no visual flash; the product price of RMB 75”. The manipulation of the experimental stimuli was measured by the question “Do you think this product is functional?” (where “1” meant strongly disagree; “7” meant strongly agree). We used this question to determine whether the subject considered the described lamp to be relatively functional or non-functional, and thus, whether the manipulation of the subject was successful.

#### 4.1.3. Purchase Intention

Regarding the measurement of purchase intention, we used Dodds et al.’s [69] scale, which measures subjects’ purchase intention through three items: “likely to consider purchasing”, “high probability of purchasing”, and “high willingness to purchase”. We used three items to measure the subjects’ purchase intention, which we used to determine the subjects’ purchase decision preferences (“1” indicated strongly disagree and “7” indicated strongly agree).

#### 4.1.4. Perfectionism

Regarding the measurement of perfectionist tendency, as in Study 1, we divided the subjects into two groups: perfectionists and non-perfectionists.

#### 4.1.5. Dichotomous Thinking

To measure dichotomous thinking, we used the dichotomous thinking questionnaire designed and developed by Byrne et al. [70] in this study. The scale contains seven questions, including “I think of things in ‘black and white’ terms”, “I think of myself as either good or bad”, and so on, to measure the tendency of dichotomous thinking. According to the mean score, the higher the score, the higher the tendency toward dichotomous thinking.

### 4.2. Results

#### 4.2.1. Manipulation Check

The results of an independent sample T-test showed that the subjects in the group considered the defective product function as significantly less functional compared with those in the group with perfect product function (M_defective function_ = 2.87, SD = 1.737 vs. M_perfect function_ = 5.87, SD = 0.937, F (1, 58) = 69.333, *p* < 0.001).

#### 4.2.2. Reliability of Scale

Then, we examined the reliability of the scales of willingness to pay (Cronbach’s alpha = 0.938, with 95% confidence intervals), perfectionism (Cronbach’s alpha = 0.876, with 95% confidence intervals), and dichotomous thinking (Cronbach’s alpha = 0.916, with 95% confidence intervals). The findings indicated that the analysis based on these scales is highly credible.

#### 4.2.3. Purchase Intention

**Figure 2:**
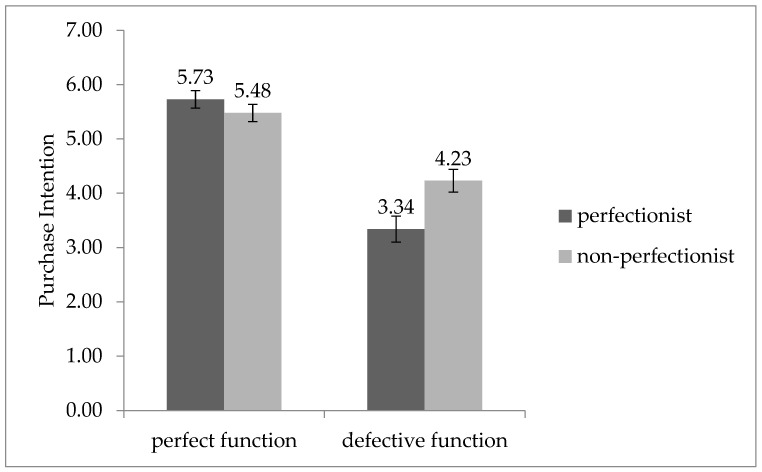
Interaction of purchase intention between perfectionism and product function.

The results of the analysis of variance (ANOVA) in Figure 2 showed that perfectionism and product function significantly interacted with consumers’ purchase intention (F (1, 134) = 8.104, *p* = 0.005 < 0.01). Specifically, consumers with perfectionism showed significantly lower intention to purchase products with defective functions than those without perfectionism (M_perfectionist_ = 3.34, SD = 1.43 vs. M_non-perfectionist_ = 4.23, SD = 1.27, F (1, 70) = 7.760, *p* = 0.007 < 0.01). However, we found no significant difference between consumers with and without perfectionism in the intention to purchase products with perfect function (M_perfectionist_ = 5.73, SD = 0.94 vs. M_non-perfectionist_ = 5.48, SD = 0.90, F (1, 64) = 1.151, *p* = 0.287 > 0.05). H1 was validated again.

#### 4.2.4. Mediation Analysis

We used the Bootstrap method to test whether dichotomous thinking played a mediating role in the effect of perfectionism on the intent to purchase imperfect products. With 5000 resamples and a 95% confidence interval, the results of the mediating effect did not contain zero (LLCI = 0.0306, ULCI = 0.6029), indicating that the mediating effect of dichotomous thinking was significant, and the mediating effect size was 0.2836. In addition, after controlling the mediating variable “dichotomous thinking”, the perfectionism of the independent variable had no significant effect on the intent to purchase imperfect products of the dependent variable, and the interval (LLCI = −0.0586, ULCI = 1.2692) contained zero. Therefore, when perfectionists buy imperfect products, their dichotomous thinking completely mediates their intent to purchase imperfect products.

### 4.3. Discussion

In Study 2, we measured the intent of perfectionists and non-perfectionists to purchase functionally perfect and functionally deficient products, separately, and tested whether dichotomous thinking played a mediating role in the main effect. The results of the obtained data once again supported H1 and validated H3. Due to the dichotomous way of thinking, consumers with perfectionist tendencies are more unacceptable of products with functional defects than those without perfectionist tendencies. Next, in Study 3, we explored whether another mediation mechanism influenced the main effect.

## 5. Study 3

We adopted appearance, another important dimension of formal imperfect products, as the experimental material of Study 3, selected agricultural products that consumers have more daily contact with as the experimental stimulus, and verified whether there was a mediating effect of intolerance of uncertainty. We believed that the risk to safety and health posed by product defects was intolerable to perfectionists and led to avoidance behavior.

### 5.1. Method

#### 5.1.1. Participants

For Study 3, we selected 140 students from a business school at a university in Beijing and distributed questionnaires during class. Of these, 5 questionnaires were considered invalid due to grouping conditions, and 3 questionnaires whose prices were three standard deviations higher than the average value: RMB 20, 20, and 30, which were also regarded as invalid questionnaires. Thus, the total number of valid questionnaires was 132 (94.3%), of which 62.12% were women and 37.88% were men. After completing the study, they were paid RMB 1.

#### 5.1.2. Procedure and Stimuli

Study 3 consisted of a 2 (perfectionist vs. non-perfectionist) × 2 (aesthetic appearance vs. flawed appearance) between-subjects design. In Study 3, we first asked subjects to read a shopping scenario: “Suppose you are choosing to buy chili peppers in the fresh produce section of a retail supermarket and the product looks as shown in the picture, please look at the picture and answer the following questions”. The aesthetic group was shown a picture of a symmetrically shaped, freshly colored pepper with no surface defects, while the flawed group was shown a picture of a misshapen, not freshly colored pepper with surface defects.

#### 5.1.3. Purchase Intention

Regarding the measurement of purchase intention, we used Ayadi and Lapeyre’s [71] measure of purchase intention in the form of price assessment, where a higher predicted price implied a higher purchase intention. We set the question “Please guess the price per 500 g of these chili peppers” to determine the subjects’ purchase intention by the high or low price they provided.

#### 5.1.4. Perfectionism

Regarding the measurement of perfectionism, as in Study 1, we divided the subjects into two groups: perfectionists and non-perfectionists.

#### 5.1.5. Intolerance of Uncertainty

Regarding the measurement of intolerance of uncertainty, we used the simplified version of the IUS scale (IUS-12), as reworked by Carleton et al. [52], in this study. The scale contains 12 items and measures subjects’ anxiety and avoidance through a two-factor structure of anticipatory anxiety and inhibitory anxiety. The scale includes questions such as “Uncertainty makes it hard for me to have a perfect life”, “I get frustrated if I don’t have all the information I expect”, and “Uncertainty stops me when it’s time to take action”.

### 5.2. Results

#### 5.2.1. Manipulation Check

We conducted a pretest study before Study 3, in which we measured whether subjects perceived the product to be defective in appearance. The results of an independent samples T-test revealed that subjects significantly perceived the peppers shown in the pictures in the flawed appearance group to have more obvious and unattractive appearance defects than the peppers shown in the pictures in the aesthetic appearance group (M_flawed appearance_ = 1.70, SD = 0.952 vs. M_aesthetic appearance_ = 5.70, SD = 1.179, F (1, 58) = 209.009, *p* < 0.001).

#### 5.2.2. Reliability of Scale

Then, we examined the reliability of the scales of perfectionism (Cronbach’s alpha = 0.856, with 95% confidence interval) and intolerance of uncertainty (Cronbach’s alpha = 0.894, with 95% confidence interval), which showed that the analysis based on these scales is highly credible.

#### 5.2.3. Purchase Intention

**Figure 3:**
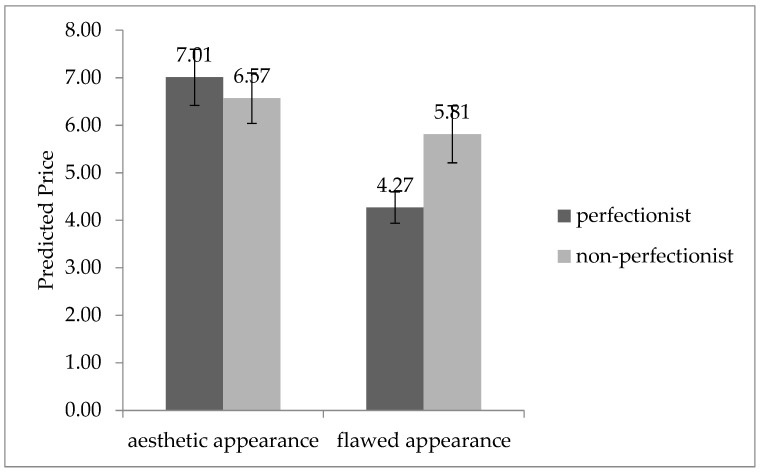
Purchase intention for products with aesthetic or flawed appearance according to perfectionists or non-perfectionists.

The results of the analysis of variance (ANOVA) in Figure 3 showed that the interaction of perfectionism and product appearance on consumers’ purchase intention was close to significant (F (1, 128) = 3.467, *p* = 0.065). Specifically, consumers with perfectionism had significantly lower intent to purchase an imperfect product with a flawed appearance than those without perfectionism (M_perfectionist_ = 4.27, SD = 1.892 vs. M_non-perfectionist_ = 5.81, SD = 3.306, F (1, 60) = 5.116, *p* = 0.027 < 0.05). However, we found no significant difference between consumers with and without perfectionism in intent to purchase the product with an aesthetic appearance (M_perfectionist_ = 7.01, SD = 3.511 vs. M_non-perfectionist_ = 6.57, SD = 3.146, F (1, 68) = 0.305, *p* = 0.582 > 0.05). H1 was validated again.

#### 5.2.4. Mediation Analysis

Next, we used the Bootstrap method to test whether intolerance of uncertainty played a mediating role in the effect of perfectionism on the intent to purchase imperfect products. With 5000 resamples and a 95% confidence interval, the results of the mediating effect did not contain zero (LLCI = 0.0563, ULCI = 1.5734), indicating that the mediating effect of intolerance of uncertainty was significant, and the mediating effect size was 0.7755. In addition, after including the mediating variable “intolerance of uncertainty”, perfectionism as the independent variable had no significant effect on the purchase intention of imperfect products as the dependent variable. The interval (LLCI = −0.8175, ULCI = 2.3366) contained zero. Therefore, when perfectionists buy products with a flawed appearance, their personality traits of intolerance of uncertainty completely mediate their intent to purchase imperfect products.

### 5.3. Discussion

Study 3 is based on Study 2 to further verify what other mediating mechanisms exist that influence the main effect. Product appearance defects are another manifestation of imperfect products and are an important factor influencing consumers’ purchase decisions. In Study 3, we measured the intentions of perfectionists and non-perfectionists to purchase flawed and aesthetic products using the appearance of products as the study stimulus. We examined whether intolerance of uncertainty mediated the main effect. The results supported H1 again and confirmed H4, i.e., that perfectionist consumers are more likely to avoid purchasing products with a flawed appearance because they are less likely to tolerate uncertainty about their health and safety than those who are not a perfectionist. We also confirmed the mediating role of intolerance of uncertainty between perfectionism and preference for imperfect products.

## 6. Study 4

Our purpose in Study 4 was to verify H2 and the mediating effects of H3 and H4, that is, whether incomplete after-sales service in imperfect products leads to stronger avoidance of product purchase in perfectionists compared with non-perfectionists, and to verify the influence of mediating factors. The findings suggested that the uncertainty of future use produced by incomplete after-sales service triggers perfectionists’ characteristics of intolerance of uncertainty, and dichotomous thinking also leads to perfectionists paying more attention to the incompleteness of after-sales service and their avoidance of purchase.

### 6.1. Method

#### 6.1.1. Participants

In Study 4, the participants were 140 university students in Beijing, of which 4 responses were considered invalid due to grouping conditions. The number of valid responses was 136 (97.1%), of which 69.12% were women and 30.88% were men. After completing the study, they were paid RMB 1.

#### 6.1.2. Procedure and Stimuli

Study 4 consisted of a 2 (perfectionist vs. non-perfectionist) × 2 (complete after-sales service vs. incomplete after-sales service) between-subjects design. In Study 4, we first asked subjects to read a shopping scenario: “Suppose you are planning to buy a cell phone shortly for daily study, work, and entertainment use. The phone is equipped with a 5G network; 8 + 128G memory; 50-megapixel triple camera system; a super retina XDR display; with the latest generation processor; and the price of the item is RMB 3500”. The after-sales service received by the complete group is described as “the product enjoys free repair within one year (for any reason); provides extended warranty options; guaranteed return and replacement within a month due to quality problems”. The incomplete group received a description of the after-sales service as follows: “the product is free of charge for repair within one year (for some reasons at your own expense); no extended warranty option; exchangeable (not refundable) within one month due to quality problems”.

#### 6.1.3. Purchase Intention

Regarding the measurement of purchase intention, as in Study 2, we conducted this study using Dodds et al.’s [69] scale.

#### 6.1.4. Perfectionism

Regarding the measurement of perfectionist tendency, as in Study 1, we divided the subjects into two groups: perfectionists and non-perfectionists.

#### 6.1.5. Dichotomous Thinking

Regarding the measurement of dichotomous thinking, as in Study 2, we conducted this study using the dichotomous thinking measurement questionnaire designed and developed by Byrne et al. [70].

#### 6.1.6. Intolerance of Uncertainty

Regarding the measure of intolerance of uncertainty, as in Study 3, we performed this measurement using a simplified version of the IUS scale (IUS-12) reworked by Carleton et al. [52].

### 6.2. Results

#### 6.2.1. Manipulation Check

In the pretest study, we measured the completeness of the after-sales service of the product in the study. The results of an independent samples T-test revealed that subjects in the incomplete after-sales service group significantly perceived the after-sales service in their group to be more incomplete than those in the complete after-sales service group (M_incomplete_ = 4.03, SD = 2.236 vs. M_complete_ = 5.74, SD = 1.137, F (1, 58) = 9.797, *p* = 0.003 < 0.01).

#### 6.2.2. Reliability of Scale

Then, we examined the reliability of the scales of willingness to pay (Cronbach’s alpha = 0.792, with 95% confidence interval), perfectionism (Cronbach’s alpha = 0.882, with 95% confidence interval), dichotomous thinking (Cronbach’s alpha = 0.856, with 95% confidence interval), and intolerance of uncertainty (Cronbach’s alpha = 0.879, with 95% confidence interval), which indicated that the analysis based on these scales is highly credible.

#### 6.2.3. Purchase Intention

The results of the analysis of variance (ANOVA) showed that the interaction of perfectionism and product after-sales service integrity on consumers’ purchase intention was significant (F (1, 132) = 5.178, *p* = 0.024). Specifically, consumers with perfectionism had significantly lower intent to purchase the imperfect product with incomplete after-sales service than those without perfectionism (M_perfectionist_ = 4.97, SD = 1.232 vs. M_non-perfectionist_ = 5.59, SD = 0.672, F (1, 68) = 6.807, *p* = 0.011 < 0.05). However, we found no significant difference between consumers with and without perfectionism in intent to purchase a product with complete after-sales service (M_perfectionist_ = 5.87, SD = 0.777 vs. M_non-perfectionist_ = 5.79, SD = 0.785, F (1, 64) = 0.177, *p* = 0.676 > 0.05). H2 was validated.

#### 6.2.4. Mediation Analysis

**Figure 4:**
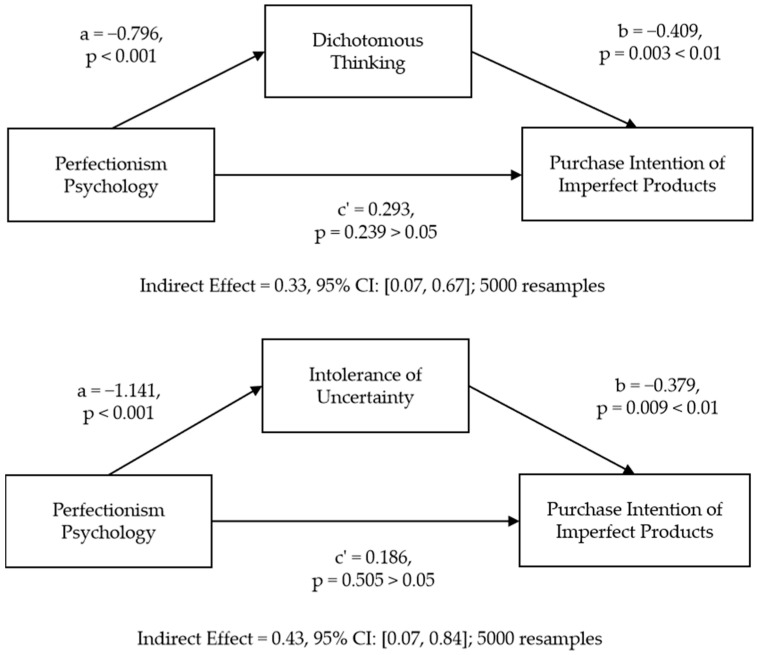
Mediating role of dichotomous thinking and intolerance of uncertainty.

Next, we conducted mediation analyses with the Bootstrap method to test the downstream effects of perfectionism (perfectionist = 1, non-perfectionist = 2) on the intent to purchase imperfect products. The results in Figure 4 showed that perfectionism increased the dichotomous thinking of consumers (a = −0.796, *p* < 0.001), which in turn reduced their intent to purchase imperfect products (b = −0.409, *p* = 0.003 < 0.01). The significant indirect effect suggested perfectionism reduces the purchase intention by increasing the dichotomous thinking of consumers (indirect effect = 0.33, 95% confidence interval (CI): [0.07, 0.67]; 5000 resamples). The direct effect of perfectionism on the intent to purchase imperfect products was not significant (c’ = 0.293, *p* = 0.239 > 0.05). The results also showed that perfectionism increased the intolerance of uncertainty of consumers (a = −1.141, *p* < 0.001), which in turn reduced the intent to purchase imperfect products (b = −0.379, *p* = 0.009 < 0.01). Therefore, we concluded that the perfectionism of consumers reduces their purchase intention by increasing their intolerance of uncertainty (indirect effect = 0.43, 95% CI: [0.07, 0.84]; 5000 resamples). The direct effect of perfectionism on the intent to purchase imperfect products was not significant (c’ = 0.186, *p* = 0.505 > 0.05).

### 6.3. Discussion

In Study 4, we explored another manifestation of the main effect by replacing the formally imperfect product with imperfect product service and continued to test whether the main effect and the mediating effect still existed. In Study 4, we used the after-sales service completeness for a cell phone as the study to measure the purchase intentions of perfectionists and non-perfectionists for products with incomplete and complete after-sales service, and to test whether dichotomous thinking and intolerable uncertainty played a mediating role in the main effect. The data obtained supported H2 and reconfirmed H3 and H4. Consumers with perfectionist tendencies also had dichotomous thinking, thinking that incomplete after-sales service does not meet their high standards and that the incomplete after-sales service poses uncertainty to future use that perfectionist consumers cannot tolerate. Therefore, they are less receptive to incomplete after-sales service than consumers who do not have perfectionist tendencies.

## 7. General Discussion

In this study, we empirically analyzed the effect of perfectionism on the choice to purchase imperfect products and explored the psychological mechanism of the effect. Through a series of studies, we found that perfectionist consumers were less willing to purchase imperfect products than non-perfectionist consumers and that perfectionism influenced consumers to avoid purchasing imperfect products that were close to their expiration dates, lacked functionality, were defective in appearance, and had incomplete after-sales service. In addition, dichotomous thinking and intolerance of uncertainty mediated this effect. We tested this effect in four experimental studies, and the results showed that this mechanism is robust across a range of possible types of imperfect products (e.g., formal vs. service) and across different dependent variables (i.e., purchase intention and willingness to pay).

### 7.1. Theoretical Implications

Although scholars have previously explored the effects of perfectionism in the marketing field [1,42,67,72,73], there is less research on the impact of perfectionism on specific consumer behaviors. In our study, we explored the impact of perfectionism on consumers’ willingness to choose imperfect products, which complements the findings on perfectionism in the field of consumer behavior research, bridging the gap between the less relevant literature in the field of perfectionism influencing consumer choice issues. In addition, we added the service factor as another manifestation of the product to the study of imperfect products, broadening the scope of the study of imperfect products.

To test the influence of perfectionism on the choice to purchase imperfect products, we proposed two possible explanations: dichotomous thinking and intolerance of uncertainty. Perfectionists have a high standard for things and, combined with a dichotomous mindset, they categorize all types of imperfect products as low-standard, which is contrary to their expectation of making optimal decisions [74], and thus, they abandon the purchase. In addition, perfectionists’ intolerance of uncertainty makes them worry about the threat to their safety and health or the uncertainty of inconvenient future use if they buy imperfect products. This study enriches our understanding of the psychological mechanisms that act when perfectionist consumers choose products.

### 7.2. Managerial Implications

In reality, certain consumer groups have a unique and firm demand for a certain type of product (e.g., a high-grade version of a camera or a mobile phone). This aroused our interest, and we found that perfectionism plays an important role in this issue. Perfectionism can be viewed as an important psychological variable and can also be used for market segmentation. Perfectionists spend more time on decision making than non-perfectionists, and perfectionists are more likely to choose products without flaws. In the context of online shopping, retailers can target these perfectionist consumers according to their past purchases and product browsing behavior, enabling retailers to make more accurate product recommendations.

Our findings also provide theoretical support for companies when developing product strategies by analyzing the psychological mechanisms of perfectionists in their purchase decisions. Companies whose target customers include perfectionists should pay special attention to minor defects in their products, ensure products do not near their end of shelf life by the time they are purchased, ensure perfect product function, and ensure products have an esthetically pleasing appearance, and scratches, extrusion, contamination, and other defects should be avoided in the product packaging to ensure the formal perfection of the products. Companies should also pay attention to the supporting services, try to use deterministic communication, and provide complete after-sales service procedures, to give perfectionists a sense of security; only by avoiding the perception of uncertainty by perfectionists can companies meet their needs through the products they buy.

## 8. Limitations and Future Research

In this study, we focused on perfectionist consumers’ choice to purchase imperfect products; however, the personality traits and behaviors of perfectionist consumers are multi-dimensional. Perfectionist consumers are more planned, organized about tasks, strict with themselves, and are more afraid of failure. These characteristics may influence their product and brand preferences and consumer choices, and thus, how these dimensions may influence consumers’ behavioral performance and purchase decisions should be explored in future research. Another important point is that the postpurchase emotional responses of perfectionist consumers may also substantially differ from those of non-perfectionist consumers, especially when products do not meet consumer expectations. These points are important areas for future research on the behavioral choices of perfectionist consumers.

## Data Availability

Not applicable.
